# Vancomycin-Induced Thrombocytopenia in a 35-Year-Old Female With Pneumonia: A Case Report

**DOI:** 10.7759/cureus.45945

**Published:** 2023-09-25

**Authors:** Frederick Gyabaah, Bhavi Trivedi, Swathi Prakash, Cyrena Petersen, Jordan Ikeler, Fatma Dihowm

**Affiliations:** 1 Internal Medicine, Texas Tech University Health Sciences Center, El Paso, USA; 2 Internal Medicine, Texas Tech University Health Sciences Center Paul L. Foster School of Medicine, El Paso, USA

**Keywords:** anti-platelet, medications, immune, thrombocytopenia, vancomycin

## Abstract

Vancomycin is one of the most empirically used antibiotics in severely ill patients in hospitalized settings. Vancomycin-induced thrombocytopenia (VITP) is a rare and potentially life-threatening complication that requires immediate recognition. Platelet destruction is largely immune-mediated and results in a precipitous drop in the platelet count over a short period of time. Most cases of VITP are drug-dependent, as discontinuation of the offending agent frequently results in a timely return to baseline to pre-exposure platelet levels. Here, we present a case of severe vancomycin-induced thrombocytopenia in a 35-year-old female with a history of multiple comorbidities who presented with pneumonia. She was undergoing treatment with vancomycin and piperacillin-tazobactam and developed thrombocytopenia within 24 hours of hospitalization. The patient was on a loading dose of 1250 mg intravenous vancomycin every 24 hours and piperacillin-tazobactam 3.375 g intravenously every six hours for presumed community-acquired pneumonia. Her other medications included ondansetron, bupropion, sertraline, tamsulosin, pantoprazole, ergocalciferol, and insulin glargine. Additionally, the patient was placed on a prophylactic dose of enoxaparin while in-patient. The patient’s thrombocytopenia resolved with discontinuation of vancomycin. Clinicians should be well-informed about which medications can trigger thrombocytopenia whenever starting a medication in such cases.

## Introduction

Drug-induced thrombocytopenia (DITP) is a well-described complication in the medical literature. It has been associated with multiple drug classes, including alkaloids, platelet inhibitors, anticonvulsants, analgesics, antimalarials, anticoagulants, and antibiotics [1]. Drugs can induce thrombocytopenia through immune-mediated or non-immune-mediated mechanisms. The pathophysiology of DITP typically involves immune-mediated destruction of platelets due to the formation of an antibody that targets a platelet membrane protein, such as glycoprotein 1b/IX/V (GP1b/IX/V) and glycoprotein IIb/IIIa (GPIIb/IIIa) [2,3].

Vancomycin is a glycopeptide, bactericidal antibiotic that inhibits cell wall synthesis in bacteria. It is empirically used to treat gram-positive bacterial infections, and usually the drug of choice for methicillin-resistant *Staphylococcus aureus* (MRSA) infections [4]. Vancomycin-induced thrombocytopenia (VITP) is a commonly overlooked side effect especially in an individual on numerous medications [5-7]. Vancomycin can cause immune-mediated thrombocytopenia by initiating the production of typically immunoglobulin M (IgM) antibodies that act through a “quinine-like” mechanism. Vancomycin binds to the Fab region of an antibody and/or membrane glycoprotein of a platelet, preferentially binding GPIIb/IIIa, which enhances the antibodies' affinity for and binding to these platelet glycoproteins. These vancomycin-dependent antibodies can then target platelets, which leads to platelet destruction and/or clearance [1,8]. This report details the events in a case of a 35-year-old female patient who presented with pneumonia and developed rapid-onset VITP within 24 hours of administration, which resolved with discontinuation of vancomycin.

## Case presentation

A 35-year-old African American female presented with complaints of diffuse abdominal pain, shortness of breath and cough with sputum. Her medical history included nonobstructive right nephrolithiasis, anxiety, type 2 diabetes mellitus, Mobitz type II heart block, gastroesophageal reflux disorder, remote cholecystectomy and a known history of MRSA colonization [9].

On admission, her white blood cell count was 13.74 x 10^3^/mm^3^ with neutrophilic predominance; the platelet count was 283 x 10^3^/mm^3^. Her serum creatinine concentration was within normal limits at 0.7 mg/dL, and no other abnormal chemistry values were noted. Her chest x-ray reported bilateral pleural effusions with bilateral lobe compressive atelectasis. The patient was given a loading dose of 1250 mg intravenous vancomycin followed by a maintenance dose of 750 mg IV every 12 hours and piperacillin-tazobactam 3.375 g intravenously every 6 hours for presumed community-acquired pneumonia. Her other medications included ondansetron, bupropion, sertraline, tamsulosin, pantoprazole, ergocalciferol, and insulin glargine. Additionally, the patient was placed on a prophylactic dose of enoxaparin while in-patient [10].

Following the administration of empiric antibiotics, the patient clinically improved. Despite the patient’s trough levels within normal limits, her baseline serum creatinine concentration rose from 0.7 to 1.0 mg/dL after 24 hours of administration. Therefore, the vancomycin dose was renally adjusted to 750 mg daily. Soon after, the patient's platelets dropped to 138 x 10^3^/mm^3^, with an estimated 51% reduction in the platelet count. With the significant drop in the platelet count, enoxaparin was discontinued after 24 hours of administration and blood samples were sent to the laboratory for manual cell count. The peripheral blood smear did not show any evidence of clumping of platelets, megaloblastic changes or schistocytes. The possibility of coagulative and DITP pathologies was also ruled out with an unremarkable family history, past medical history, and negative serotonin-release assay, platelet factor 4 (PF-4) antibody, and disseminated intravascular coagulation (DIC) panel [11].

**Figure 1:**
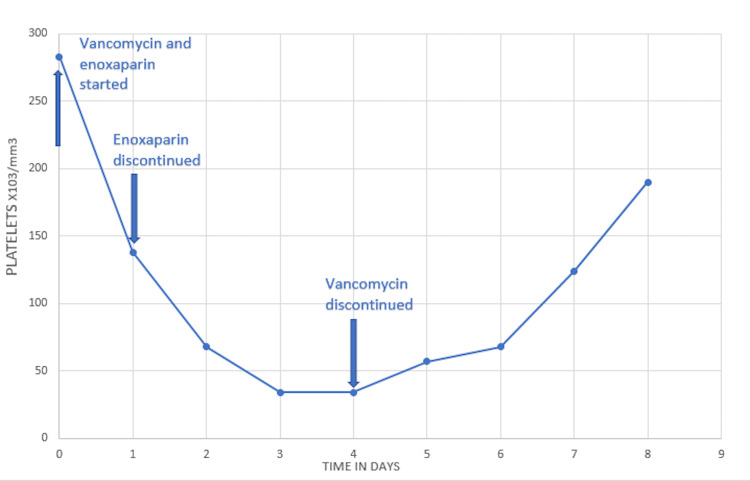
Clinical course of the patient with vancomycin-induced thrombocytopenia

Despite discontinuation of enoxaparin and a negative serotonin-release assay, the platelet levels further decreased to 34 x 10^3^/µL without any clinically significant bleeding episodes. Vancomycin was finally stopped on day 4 following which the platelet count increased from 34 x 10^3^/µL to 67 x 10^3^/µL three days after discontinuation. Her platelet count continued to improved steadily without any steroids (Figure 1). The patient’s thrombocytopenia resolved with a presumptive diagnosis of VITP.

## Discussion

The presumptive diagnosis of vancomycin-induced thrombocytopenia is a type of drug-induced thrombocytopenia, based on clinical criteria and laboratory studies. Many medications, including alkaloids, platelet inhibitors, anticonvulsants, analgesics, antimalarials like quinine, anticoagulants like heparin, and antibiotics like vancomycin, can induce thrombocytopenia [1,2]. Therefore, when a patient is on one of these medications and has a drop in the platelet count, there is an increased clinical suspicion of DITP.

The International Society on Thrombus and Hemostasis (ISTH) released recommendations on DITP laboratory testing, which is required to confirm the diagnosis [1]. Enzyme immunoassay and flow cytometry are the two major methods to determine the presence of drug-dependent antibodies. A positive test correlates with a high specificity for the presence of drug-dependent platelet antibodies. If a patient has a positive result, they should avoid exposure to that specific drug in the future [5]. However, laboratory testing is not widely utilized due to the lack of availability and the time taken (up to several days) for these tests to be completed; therefore, the clinical criteria are an important factor in determining the likelihood of DIPT before detrimental side effects begin.

In a study published in the *New England Journal of Medicine*, platelet-reactive antibodies of IgG, IgM, or both classes with vancomycin dependency were reported in 20% of the samples referred for vancomycin-induced thrombocytopenia testing [10]. A mean nadir platelet count of 13,600 per cubic millimeter (ranging from 1000 to 60,000) was attained after about eight days (ranging from 1 to 27 days) after vancomycin initiation [11]. Platelet levels returned to baseline in the median time of 7.5 days. Of note, one patient in the study had prolonged thrombocytopenia, which was attributed to renal insufficiency slowing the clearing of vancomycin out of the system [12]. Additionally, previous studies have found no statistically significant correlation between vancomycin levels and the severity of thrombocytopenia [13].

Our patient was started on vancomycin prior to the onset of the thrombocytopenia and recovered after vancomycin was discontinued [14]. Additionally, there is a high likelihood that she was previously exposed to vancomycin for the treatment of an MRSA infection that was noted in her past medical history. This previous exposure was most likely the catalyst for the formation of vancomycin-dependent antibodies. Following the re-administration of vancomycin, the patient underwent a severe reaction with a drop in platelets that began to rise after the discontinuation of vancomycin. This course of events fits the clinical criteria for the diagnosis of drug-induced thrombocytopenia despite the unavailability of antibody testing.

Thrombocytopenia is defined as a platelet count less than 150 x 10^3^/µL for adults. Decreased platelet counts increase the risk of bleeding, which can include minor bleeds such as epistaxis or mucosal bleeds. However, it can also include more severe forms of bleeding, such as internal or external hemorrhaging, which can be life-threatening and must be monitored and addressed in an efficient manner [15]. Platelets are an essential component for primary hemostasis. Platelets aggregate at the site of a bleed and bind to von Willebrand factor and fibrinogen to form a thrombus to inhibit bleeding. A reduction in platelet count inhibits this pivotal mechanism of thrombosis [16].

DITP can be difficult to diagnose as the patients may not present with clinical symptoms until later in the disease process [17]. Therefore, it is important to monitor the platelet count and watch for the presence of petechiae, purpura, and other signs of bleeding when initiating a medication that has been associated with DITP. Treatment usually involves discontinuation of the suspected offending agent and implementation of bleeding precautions for the patient [18].

## Conclusions

This case report detailed the clinical course of a 35-year-old female who presented with pneumonia and developed DITP in response to the administration of vancomycin within 24 hours of vancomycin administration. Vancomycin was discontinued and the patient’s platelet count returned to baseline after five to seven days. This suggests the relationship between vancomycin-induced platelet reactive antibodies of the IgG, IgM class and platelet turnover. DITP is an easily missed diagnosis, but is important to recognize because it can have an insidious onset and the use of medications that can induce thrombocytopenia, such as vancomycin, are commonplace in the hospital setting. The consequences that can result from DITP are life-threatening, such as internal or external hemorrhaging, further highlighting the importance of recognizing this diagnosis early in its onset. DIPT can present clinically with signs of bleeding, such as petechiae or purpura; however, patients may be asymptomatic, like our patient. It is important for clinicians to appropriately perform a physical examination to assess physical manifestations of thrombocytopenia, and laboratory testing to monitor decreased platelet counts whenever starting a medication with a known association with thrombocytopenia. Clinicians should be well informed of which medications have been correlated to DITP, so that severe complications can be detected and treated on an urgent basis. Finally, time to recovery after the discontinuation of vancomycin depends on the individual platelet turnover, which is the function of effective hematopoiesis. In this case, recovery of patient's platelet count to the pre-hospitalization baseline took about a week after the discontinuation of the offending agent (vancomycin).
